# In Vitro Antimicrobial Susceptibility of *Mycobacterium massiliense* Recovered from Wound Samples of Patients Submitted to Arthroscopic and Laparoscopic Surgeries

**DOI:** 10.1155/2011/724635

**Published:** 2011-01-04

**Authors:** Alessandra Marques Cardoso, Ana Paula Junqueira-Kipnis, André Kipnis

**Affiliations:** Departamento de Microbiologia, Imunologia, Parasitologia e Patologia, Instituto de Patologia Tropical e Saúde Pública, Universidade Federal de Goiás, Rua Delenda Rezende Melo com 1 Avenida, S/N, Setor Universitário, Goiânia, 74605-050 Goiás, Brazil

## Abstract

Testing of rapidly growing species of mycobacteria (RGM) against antibacterial agents has been shown to have some clinical utility. This work establishes the MICs of seven antimicrobial agents following the guidelines set forth by the Clinical and Laboratory Standards Institute (CLSI) against eighteen isolates of *Mycobacterium massiliense* recovered from wound samples of patients submitted to minimally invasive surgery such as arthroscopy and laparoscopy. The isolates showed susceptibility to amikacin (MIC_90_ = 4 *μ*g/mL) and clarithromycin (MIC_90_ < 1 *μ*g/mL) but resistance to ciprofloxacin (MIC_90_ > 16 *μ*g/mL), doxycycline (MIC_90_ > 32 *μ*g/mL), sulfamethoxazole (MIC_90_ > 128 *μ*g/mL), and tobramycin (MIC_90_ = 32 *μ*g/mL), and intermediate profile to cefoxitin (MIC_90_ = 64 *μ*g/mL). Therefore, we suggest that the antimicrobial susceptibilities of any clinically significant RGM isolate should be performed.

## 1. Introduction

Infections by rapidly growing mycobacteria (RGM) are increasing in minimally invasively surgeries worldwide [1–3]. *Mycobacterium massiliense* has been isolated from pacemaker pocket infection, intramuscular injections, and post-video surgical infections [1, 2, 4–6]. *Mycobacterium massiliense* was validated as a separate species from the *M. chelonae abscessus *group in 2004 [4]. 

In Brazil, outbreaks caused by RGM have been reported since 1998. The former outbreaks occurred following laser in situ keratomileusis (surgery for myopia correction), mesotherapy sessions (intradermal injections) or breast implants. Likewise, in those outbreaks *M. chelonae-abscessus *group was the main pathogen found [7, 8]. Recently, an epidemic of surgical-site infections was reported in seven different regions of Brazil, and surprisingly it was shown to be caused by a single clone of *M. massiliense* [1, 2, 9, 10]. 

RGM are intrinsically resistant to several antibiotic drugs reducing the number of active drugs to treat infections by these bacteria and therefore antimicrobial susceptibility testing have been shown to improve the clinical outcome [11–13]. For this reason, it is recommended that all clinically significant isolates should be tested against selected antimicrobial agents [14, 15].

The Clinical and Laboratory Standards Institute (CLSI) recommends the standard broth microdilution method for susceptibility testing of the *Mycobacterium fortuitum* group (*M. fortuitum, M. peregrinum, *and *M. fortuitum* third variant complex), *Mycobacterium chelonae*, and *Mycobacterium abscessus. *The method and guidelines for interpretation of results, on theoretical grounds, also should apply to *Mycobacterium mucogenicum*, *Mycobacterium smegmatis* group (*M. smegmatis, M. goodii, *and *M. wolinskyi*), and the clinically significant, pigmented RGM; however, data to support this are not currently available [16]. Being a recently classified RGM, *M. massiliense* susceptibility testing guidelines are needed, and susceptibility testing data from different settings will contribute for this goal. 

The aim of this work was to examine the in vitro susceptibilities of *M. massiliense *isolates from wound samples of patients with infection post-video surgeries such as arthroscopy and laparoscopy at seven private hospitals of Goiânia, Goiás, Brazil.

## 2. Materials and Methods

### 2.1. Mycobacterial Strains

The patients were from seven private hospitals in the city of Goiânia, State of Goiás, Brazil. Patient enrollment occurred between August 2005 and July 2007. Eighteen epidemic isolates of *M. massiliense* were included in this study, after patients signed consent agreement. The microorganisms were isolated from clinical samples of 18 patients that presented signs and symptoms of localized infection after minimally invasive surgery (arthroscopy or laparoscopy). *M. massiliense *strains were identified to the species level by PCR-restriction digestion of the *hsp65* gene, pulsed-field gel electrophoresis (PFGE) comparisons, and *rpo*B partial gene sequencing [1]. Isolates were maintained on Lowenstein-Jensen slants prior to being tested and subcultured onto Mueller-Hinton plates at 35°C for 3 to 5 days. *M. abscessus *ATCC 19977 was used as a quality control strain. 

### 2.2. Antimicrobial Agents and Microdilution Trays

Serial twofold dilutions of antimicrobial solutions were added to Mueller-Hinton broth to achieve final concentrations of antimicrobial agents (all from Sigma Aldrich, USA): amikacin (2 to 128 *μ*g/mL), cefoxitin (4 to 256 *μ*g/mL), ciprofloxacin (0,25 to 16 *μ*g/mL), clarithromycin (1 to 64 *μ*g/mL), doxycycline (0,5 to 32 *μ*g/mL), sulfamethoxazole (2 to 128 *μ*g/mL), and tobramycin (0,5 to 32 *μ*g/mL) and added across the 96-well plates. 

### 2.3. Susceptibility Testing

Minimal inhibitory concentrations (MICs) of all tested drugs were determined by the broth microdilution method according to the guidelines described by the CLSI [16]. The final inoculum size was between 10^4^ and 10^5^ CFU/mL. Inoculated plates were sealed inside plastic bags and incubated at 35°C. All tests were performed in triplicate and the MICs were read at 72 h. The susceptibility categories of all antimicrobial agents were determined according to the breakpoints recommended by CLSI [16].

## 3. Results and Discussion

Eighteen isolates of *M. massiliense* were recovered from wound samples of patients submitted to minimally invasive surgery such as arthroscopy (*n* = 14, 77.8%) and laparoscopy (*n* = 4, 22.2%). 

All 18 strains tested were susceptible to amikacin (MIC_90_ = 4 *μ*g/mL) and clarithromycin (MIC_90_< 1 *μ*g/mL) but resistant to ciprofloxacin (MIC_90_> 16 *μ*g/mL), doxycycline (MIC_90_> 32 *μ*g/mL), sulfamethoxazole (MIC_90_> 128 *μ*g/mL), and tobramycin (MIC_90_ = 32 *μ*g/mL). All isolates had intermediate MICs for cefoxitin (MIC_90_ = 64 *μ*g/mL). The results are summarized in Table 1.

 RGM are intrinsically resistant to the antibiotics used for tuberculosis treatment, consequently patients treatment with antituberculosis drugs for infection with those bacteria may become compromised [17]. Infections by RGM that have adequately and early identification of the microorganism will have a better result outcome [18]. Susceptibility testing is a powerful tool in order to point for the use of the most effective drug and consequently enhancing the treatment success rate. 

Our susceptibility profile results corroborate with those of Adékambi et al. [4], except for the elevated MIC values encountered for doxycycline. In regard to this particular drug, other groups have reported similar findings [2, 6, 9]. This finding strengthen the idea that doxycycline should not be used as a differentiation marker between *M. massiliense* and *M. abscessus* [4]. 

According to CLSI [16], results for tobramycin should not be reported for the *M. fortuitum *or *M. abscessus* groups, as the treatment with this drug will only be superior to amikacin in infections caused by *M. chelonae*. No susceptibility testing recomendation is avaliable for *M. massiliense* yet but, according to our data, this drug should not be used, as all isolates were resistant to tobramycin. We have encountered 100% of resistance among the strains tested for sulfamethoxazole, similarly to the susceptibility reported for *M. chelonae *e *M. abscessus *[16].

Clarithromycin has been indicated as the first-line drug of choice for the treatment of infections caused by RGM [14, 19] and is appropriate for *M. massiliense* as well, as all of the strains tested for this drug in our study were susceptible. Koh et al. (2010) compared treatment outcomes of patients infected with either *M. abscessus* or *M. massiliense* and concluded that treatment response rates to combination antibiotic therapy including clarithromycin were much higher in patients with *M. massiliense* than in those with *M. abscessus* lung disease [20]. 

Acquired resistance to clarithromycin has been reported with a rate of 2.3% in patients receiving monotherapy [21] and one death have been reported due to infection with a resistant strain [22]. A previous monotherapy trial of clarithromycin for cutaneous disease caused by *M. chelonae *in immunosuppressed patients (all patients were on corticosteroids) resulted in acquired resistance among isolates from 1 of 10 (10%) patients with disseminated disease and none of 4 (0%) patients with localized disease [23]. Because of the risks relative to resistance development, it has been recommended the association of a second drug in the treatment for infections with these bacteria. Amikacin seems a good candidate, as in our study all strains were susceptible to this drug.

Another drug that is associated with clarithromycin to treat infections with RGM is cefoxitin [24], however our results showed that *M. massiliense* isolates presented an intermediary susceptibility to this drug. The different profile of susceptibilities found in our study and others stress the need for the proper RGM identification followed by a drug susceptibility screening in order to provide the most appropriate antibiotic treatment. 

The treatment of serious infections with RGM is a problem and limited by the small number of available drugs with activity at clinically achievable levels in tissue or/and blood. Each species and strain must be individually evaluated, and it is advisable always to perform in vitro sensitivity tests before using the drug for human therapy [25].

## 4. Conclusions

In conclusion, this study found that the MICs were higher for *M. massiliense *when tested cefoxitin, ciprofloxacin, doxycycline, sulfamethoxazole and tobramycin. Therefore, amikacin and clarithromycin were active against *M. massiliense *strains isolated in our study.

## Figures and Tables

**Table 1:** Antimicrobial susceptibility of 18 *Mycobacterium massiliense* strains recovered from wound infections after arthroscopic and laparoscopic surgeries during an outbreak in Goiânia, Goiás, Brazil.

Antimicrobial	MIC_50_	MIC_90_	%
Amikacin	<2	4	100 (S)
Cefoxitin	32	64	100 (I)
Ciprofloxacin	>16	>16	100 (R)
Clarithromycin	<1	<1	100 (S)
Doxycycline	>32	>32	100 (R)
Sulfamethoxazole	>128	>128	100 (R)
Tobramycin	>32	>32	100 (R)

All results were determined by broth microdilution method; I: Intermediate; R: resistant; S: susceptible; CIM_50_: the MIC capable of preventing the growth of 50% of the isolates; MIC_90_: the MIC capable of preventing the growth of 90% of the isolates.

## References

[1] Cardoso AM, Martins de Sousa E, Viana-Niero C (2008). Emergence of nosocomial *Mycobacterium massiliense* infection in Goiás, Brazil. *Microbes and Infection*.

[2] Duarte RS, Lourenço MCS, Fonseca LDS (2009). Epidemic of postsurgical infections caused by *Mycobacterium massiliense*. *Journal of Clinical Microbiology*.

[3] Carbonne A, Brossier F, Arnaud I (2009). Outbreak of nontuberculous mycobacterial subcutaneous infections related to multiple mesotherapy injections. *Journal of Clinical Microbiology*.

[4] Adékambi T, Reynaud-Gaubert M, Greub G (2004). Amoebal coculture of “*Mycobacterium massiliense*” sp. nov. from the sputum of a patient with hemoptoic pneumonia. *Journal of Clinical Microbiology*.

[5] Kim HY, Yun YJ, Park CG (2007). Outbreak of *Mycobacterium massiliense* infection associated with intramuscular injections. *Journal of Clinical Microbiology*.

[6] Simmon KE, Pounder JI, Greene JN (2007). Identification of an emerging pathogen, *Mycobacterium massiliense*, by rpoB sequencing of clinical isolates collected in the United States. *Journal of Clinical Microbiology*.

[7] Freitas D, Alvarenga L, Sampaio J (2003). An outbreak of *Mycobacterium chelonae* infection after LASIK. *Ophthalmology*.

[8] Sampaio JLM, Chimara E, Ferrazoli L (2006). Application of four molecular typing methods for analysis of *Mycobacterium fortuitum* group strains causing post-mammaplasty infections. *Clinical Microbiology and Infection*.

[9] Viana-Niero C, Lima KVB, Lopes ML (2008). Molecular characterization of *Mycobacterium massiliense* and *Mycobacterium bolletii* in isolates collected from outbreaks of infections after laparoscopic surgeries and cosmetic procedures. *Journal of Clinical Microbiology*.

[10] Leão SC, Viana-Niero C, Matsumoto CK (2010). Epidemic of surgical-site infections by a single clone of rapidly growing *Mycobacteria* in Brazil. *Future Microbiology*.

[11] Swenson JM, Wallace RJ, Silcox VA, Thornsberry C (1985). Antimicrobial susceptibility of five subgroups of *Mycobacterium fortuitum* and *Mycobacterium chelonae*. *Antimicrobial Agents and Chemotherapy*.

[12] Wallace RJ, Swenson JM, Silcox VA, Bullen MG (1985). Treatment of nonpulmonary infections due to *Mycobacterium fortuitum* and *Mycobacterium chelonei* on the basis of in vitro susceptibilities. *Journal of Infectious Diseases*.

[13] Park S, Kim S, Park EM (2008). In vitro antimicrobial susceptibility of *Mycobacterium abscessus* in Korea. *Journal of Korean Medical Science*.

[14] Griffith DE, Aksamit T, Brown-Elliott BA (2007). An official ATS/IDSA statement: diagnosis, treatment, and prevention of nontuberculous mycobacterial diseases. *American Journal of Respiratory and Critical Care Medicine*.

[15] Daley CL, Griffith DE (2002). Pulmonary disease caused by rapidly growing mycobacteria. *Clinics in Chest Medicine*.

[16] CLSI (2003). Susceptibility Testing of Mycobacteria, Nocardiae, and Other Aerobic Actinomycetes. Approved Standard. *CLSI Document M24-A*.

[17] Eid AJ, Berbari EF, Sia IG, Wengenack NL, Osmon DR, Razonable RR (2007). Prosthetic joint infection due to rapidly growing mycobacteria: report of 8 cases and review of the literature. *Clinical Infectious Diseases*.

[18] Pring M, Eckhoff DG (1996). *Mycobacterium chelonae* infection following a total knee arthroplasty. *Journal of Arthroplasty*.

[19] Brown-Elliott BA, Wallace RJ (2002). Clinical and taxonomic status of pathogenic nonpigmented or late-pigmenting rapidly growing mycobacteria. *Clinical Microbiology Reviews*.

[20] Koh WJ, Jeon K, Lee NY Clinical significance of differentiation of *Mycobacterium massiliense* from *Mycobacterium abscessus*.

[21] Wallace RJ, Meier A, Brown BA (1996). Genetic basis for clarithromycin resistance among isolates of *Mycobacterium chelonae* and *Mycobacterium abscessus*. *Antimicrobial Agents and Chemotherapy*.

[22] Sanguinetti M, Ardito F, Fiscarelli E (2001). Fatal pulmonary infection due to multidrug-resistant *Mycobacterium abscessus* in a patient with cystic fibrosis. *Journal of Clinical Microbiology*.

[23] Wallace RJ, Tanner D, Brennan PJ, Brown BA (1993). Clinical trial of clarithromycin for cutaneous (disseminated) infection due to *Mycobacterium chelonae*. *Annals of Internal Medicine*.

[24] Scholze A, Loddenkemper C, Grünbaum M, Moosmayer I, Offermann G, Tepel M (2005). Cutaneous *Mycobacterium abscessus* infection after kidney transplantation. *Nephrology Dialysis Transplantation*.

[25] Cavusoglu C, Soyler I, Akinci P (2007). Activities of Linezolid against nontuberculous mycobacteria. *New Microbiologica*.

